# Evidence that dysplasia related microRNAs in Barrett’s esophagus target PD-L1 expression and contribute to the development of esophageal adenocarcinoma

**DOI:** 10.18632/aging.103634

**Published:** 2020-09-09

**Authors:** Juanjuan Xu, Zhongyuan Yin, Lin Yang, Feng Wu, Jinshuo Fan, Qi Huang, Yang Jin, Guanghai Yang

**Affiliations:** 1 Department of Respiratory Medicine, Union Hospital, Tongji Medical College, Huazhong University of Science and Technology, Wuhan 430022, China; 2 Cancer Center, Union Hospital, Tongji Medical College, Huazhong University of Science and Technology, Wuhan 430022, China; 3 Oncology Department, Tongji Hospital, Tongji Medical College, Huazhong University of Science and Technology, Wuhan 430030, China; 4 Department of Thoracic Surgery, Union Hospital, Tongji Medical College, Huazhong University of Science and Technology, Wuhan 430022, China

**Keywords:** microRNAs, PD-L1, Barrett’s esophagus, esophageal adenocarcinoma

## Abstract

Esophageal adenocarcinoma (EAC) is the cancer arising from the esophagus, which frequently develop from Barrett’s esophagus (BE). Extracellular vesicles (EVs), particularly exosomes, are nanosized vesicles of endosomal origin released from various types of cells that have been implicated in cancers. However, the significance of circulating exosomes during the progression of BE to EAC remains unknown. Sera exosmal microRNAs were profiled from 13 EAC and 12BE patients compared to 12 healthy controls. We found a substantial dysregulation of exosomal miRNA levels in BE compared to healthy control, and identified a unique signature of 24 up regulated and 14 down regulated miRNAs. Further validation showed exosomal miR-196a, -26b, -21, and -143 expression was significantly higher in BE and continued to have higher levels in EAC compared to healthy controls; while sera exosomal miR-378, -210, -205, and -200c-3p were significantly lower expressed in BE patients compared to compared to controls. Further, miR-378, -210, -205, and -200c-3p continue to have even lower levels in EAC patients compared to BE. Interestingly, sera expression levels of exosomal miR-15a, -16, and -193a-3p were significantly down regulated in BE PD-L1(+) patients; Sera exosomal miR-15a, -15b, -16, and -193a-3p expression levels in EAC PD-L1(+) patients were significantly lower (all p < 0.01) when compared to EAC PD-L1(-) patients. More importantly, the BE-EAC group had longitudinally decreased exosomal expression levels of miR-15a, -15b, -16, and -193a-3p from BE status to their EAC progression. In conclusion, distinct microRNA expression patterns were demonstrated in circulating exosomes from Barrett’s esophagus and esophageal adenocarcinoma; Furthermore exosomal microRNAs potentially targeting PD-L1 mRNA were down regulated in PD-L1 (+) BE and EAC patients.

## INTRODUCTION

Esophageal adenocarcinoma (EAC) is the cancer arising from the esophagus. EAC is also known as one of the most common types of tumors among the esophageal cancers. Barrett’s esophagus (BE) is one kind of intestinal metaplasia in the distal esophagus and also a precursor cause of EAC [1]. BE is more common in developed countries, affecting 2% of the general adult population [2]. One study with a cohort of 961 patients undergoing colonoscopy who were offered an additional endoscopy, and found an overall prevalence of 6.8%, with 5.5% for short-segment BE in persons aged 40 years or older [3]. In another similar colonoscopy-based study of 300 patients over the age of 65 years, the prevalence was 4% and 15% for long- and short-segment BE, respectively [4]. Therefore BE is common in older men and women undergoing screening colonoscopy regardless of reflux symptoms [4]. In other population-based studies, the prevalence of BE in the general population ranged between 1.3%, 1.6% and 1.9% [5–7]. The length of BE is greater in men than in women, but other features are similar [8]. Incidence rates for high-grade dysplasia/cancer are similar in men and women, although the number of cases is small [8]. BE is clinically linked to the development of EAC by a 0.5% risk each year. Dysplasia is now used as the major biomarker to classify patients with barrett’s esophagus at high risk to develop EAC [9, 10]. The transition from BE to EAC was implicated as a progress through low grade to high-grade dysplasia [11, 12]. BE included non-dysplastic Barrett’s intestinal metaplasia, low-grade and high-grade dysplasia [9, 13]. The malignant progression rate varies according to the presence of dysplasia in esophagus [9, 10, 13]. However, it remains unclear that how BE mediated dysplasia contributes to EAC development.

Among the esophagus cancer patients PD-L1 is reported to be over expressed nearly 40% and associated with the worse overall survival [14]. In clinical trials, immune check point inhibitors reported to have a response rate 9.9-33.3% in esophagus cancer [15]. Our group recently reported the identification of biomarkers, microRNAs, to determine which patients are more likely to response PD-L1 inhibitor treatment [16]. Recent studies indicated that exosomes (~50-200nm), membrane-bound extracellular vesicles, coordinate intercellular communication between different cells [17]. Exosomes could potentially regulate cancer progression and metastasis by transferring DNA, RNA or proteins between various cells in the local and distant sites [18]. MicroRNAs (miRNAs) are small non-coding RNAs that involved in messenger RNA degradation and translation [19], which also implicated in its ability to inhibit translation of tumor associated genes [20–23]. Numerous documents have implicated in miRNAs’ significance on development of BE as well as EAC [24–27], which further demonstrated the potential of miRNA profiling to distinguish BE from EAC [28–30]. However, circulating exosomal miRNAs during the disease progression of BE to EAC has yet been examined. We, therefore, aimed to characterize miRNA content of exosomes in both BE and EAC patients, further validated that circulating exosomes contain differential level of microRNAs targeting Programmed death-ligand 1 (PD-L1) mRNA in Barrett’s esophagus and esophageal adenocarcinoma.

## RESULTS

### Serum miRNAs were differentially expressed in BE and EAC

**Figure 1:**
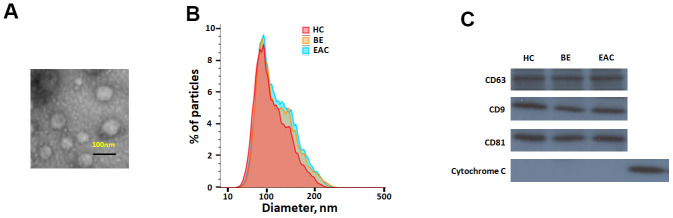
Characterisation of exosome-like vesicles released from serum by (**A**) transmission electron microscopy, (**B**) dynamic light scattering analysis (133±25 nm) and (**C**) western blotting. Presence of exosomal markers, CD63, CD9, CD81 and mitochondrial protein cytochrome c in lysates from sera-derived exosomes and cell lysate.

Sera exosomes isolated from BE and EAC were firstly characterized. Isolated exosomes were shown a spherical morphology with an average size of 133 ±25 nm (Figure 1A, 1B). Exosomal markers, CD63, CD9 and CD81 were present (Figure 1C), which clearly indicate that this vesicles are mostly exosomes. No significant differences were seen among healthy subjects, BE, and EAC.

**Figure 2:**
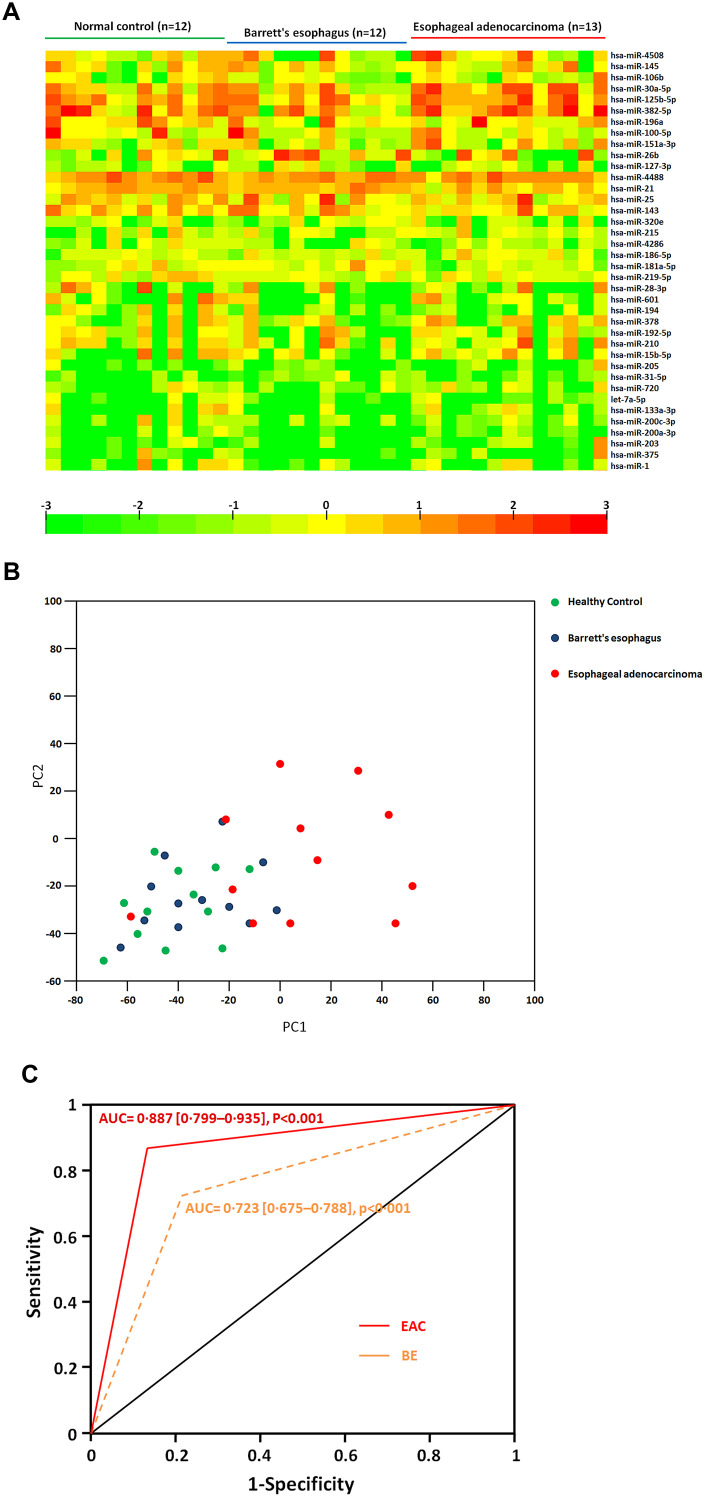
(**A**) Heatmap of differential sera miRNA profiles during the sequential progression of Barrett’s esophagus to esophageal adenocarcinoma. Heatmap representation of the mean fold change in differential miRNA signature. Two-dimensional grid matrix displaying 38 serum miRNAs was obtained by the functional heat-map in R. Columns refer to time course comparison: 12 healthy controls, 12 BE and 13 EAC. Rows stand for the 38 differential miRNAs. Each entry of the grid refers to relative fold (log2) of a given miRNA. The color of each entry is determined by the value of that fold difference, ranging from green (negative values) to red (positive values). (**B**) Principal component analysis. The plots for disease phenotypes (12 healthy controls, 12 BE and 13 EAC) were performed as principal component analysis among all samples based on miRNA profiles. (**C**) The performance of sera exosomal microRNA classifier to detect the risk of BE and EAC. ROC curves from 12 healthy controls, 12 BE and 13 EAC. Performance shown for distinguishing individuals with BE or EAC from healthy controls.

A pilot microRNA profiling was further performed to identify the differentially expressed exosome miRNAs in 13 EAC and 12BE patients compared to 12 healthy controls. Comparison shown that 38 exosomal miRNAs were differentially expressed in EAC patients compared to those of BE patients. Among them, we found that 24 exosomal miRNAs were significantly up regulated (including miR-4508, miR-145, miR-106b, miR-30a-5p, miR-125b-5p, miR-382-5p, miR-196a, miR-100-5p, miR-151a-3p, miR-26b, miR-127-3p, miR-4488, miR-21, miR-25, miR-143, miR-320e, miR-215, miR-4286, miR-186-5p, miR-181a-5p, miR-219-5p, miR-28-3p, miR-601, miR-194) and 14 miRNAs (miR-378, miR-192-5p, miR-210, miR-15b-5p, miR-205, miR-31-5p, miR-720, let-7a-5p, miR-133a-3p, miR-200c-3p, miR-200a-3p, miR-203, miR-375, miR-1) were down regulated significantly (fold change>2, p<0.05) in sera exosomes of EAC patients with compared to BE (Table 1 and Figure 2A). Moreover, principal component analysis (PCA) were performed for Healthy, BE and EAC based on miRNA profiles (Figure 2B). EAC was correlated with the first component (p<0.01). These results indicated that aberrantly expressed serum exosomal miRNA profiles were implicated in BE to EAC progression. We further identified which biologic pathways were affected during the sequential progression of BE to EAC, we applied DIANA-mirPath on the dysregulated serum miRNAs, and 52 KEGG pathways were significantly enriched (p<0.05) after false discovery rate was corrected. Fatty acid biosynthesis (2.238E-12), Hippo signaling pathway (2.604E-06), ErbB signaling pathway (1.001E-04) were top ranked as the most prominent pathways enriched in quantiles with the serum miRNA signature (Supplementary Table 1), suggesting that these biologic pathways were involved in the sequential progression of BE to EAC.

**Table 1:** Differential microRNAs expression in serum during the sequential progression of Barrett’s esophagus to esophageal adenocarcinoma.

**Barrett’s esophagus vs. Healthy Control**			**Esophageal adenocarcinoma vs. Barrett’s esophagus**		
**miR-Name**	**Fold change**	**Adjusted p-value**	**miR-Name**	**Fold change**	**Adjusted p-value**
miR-4508	2.962	0.0700	miR-4508	4.643	0.0022
miR-145	2.928	0.0650	miR-145	4.063	0.0030
miR-106b	0.344	0.0755	miR-106b	3.125	0.0071
miR-30a-5p	1.123	0.0729	miR-30a-5p	2.881	0.0015
miR-125b-5p	1.244	0.0784	miR-125b-5p	2.811	0.0016
miR-382-5p	1.254	0.0144	miR-382-5p	2.668	0.0030
miR-196a	1.153	0.0446	miR-196a	2.651	0.0031
miR-100-5p	1.166	0.0560	miR-100-5p	2.585	0.0028
miR-151a-3p	1.099	0.0740	miR-151a-3p	2.534	0.0025
miR-26b	1.186	0.0460	miR-26b	2.495	0.0056
miR-127-3p	1.186	0.0536	miR-127-3p	2.483	0.0035
miR-4488	1.067	0.0513	miR-4488	2.429	0.0006
miR-21	1.207	0.0363	miR-21	2.422	0.0019
miR-25	0.963	0.0015	miR-25	2.408	0.0043
miR-143	1.224	0.0396	miR-143	2.334	0.0038
miR-320e	0.690	0.0615	miR-320e	2.245	0.0028
miR-215	1.211	0.0550	miR-215	2.214	0.0024
miR-4286	1.085	0.0715	miR-4286	2.209	0.0033
miR-186-5p	2.077	0.0731	miR-186-5p	2.172	0.0031
miR-181a-5p	1.435	0.0523	miR-181a-5p	2.168	0.0025
miR-219-5p	0.866	0.0880	miR-219-5p	2.153	0.0065
miR-28-3p	1.415	0.0206	miR-28-3p	2.114	0.0008
miR-601	1.245	0.0183	miR-601	2.083	0.0063
miR-194	0.819	0.0190	miR-194	2.065	0.0036
miR-378	0.731	0.0358	miR-378	0.493	0.0021
miR-192-5p	0.653	0.0517	miR-192-5p	0.393	0.0032
miR-210	0.584	0.0437	miR-210	0.250	0.0012
miR-15b-5p	0.837	0.0597	miR-15b-5p	0.162	0.0041
miR-205	0.630	0.0642	miR-205	0.093	0.0015
miR-31-5p	7.275	0.0558	miR-31-5p	0.093	0.0020
miR-720	1.678	0.0551	miR-720	0.092	0.0034
let-7a-5p	1.407	0.0344	let-7a-5p	0.089	0.0033
miR-133a-3p	2.288	0.0663	miR-133a-3p	0.088	0.0065
miR-200c-3p	0.558	0.0691	miR-200c-3p	0.087	0.0034
miR-200a-3p	0.766	0.0563	miR-200a-3p	0.073	0.0021
miR-203	0.807	0.0274	miR-203	0.053	0.0024
miR-375	0.783	0.0464	miR-375	0.048	0.0005
miR-1	0.521	0.0556	miR-1	0.033	0.0032

We further identified and assessed a miRNA combination that could classify BE or EAC from healthy controls. A miRNA classifier based on the differential miRNA pattern was built to classify BE and EAC patients and from healthy individuals. The miRNA classifier showed higher accuracy to distinguish individuals with BE and EAC from controls (EAC vs. Controls, area under the curve (AUC) = 0·887 [0·799–0·935]; BE vs. Controls AUC = 0·723 [0·675–0·788], p<0·001) (Figure 2C). The miRNA classifier had higher sensitivity (range 75·4-88·9%) and specificity (82·1-93·4%) to detect EAC. These data further supported a notion that the potential mechanisms regulated by differentially expressed microRNAs would play important roles during BE and EAC development.

### Validation of dysregulated miRNAs with independent larger sample-size cohort

**Figure 3:**
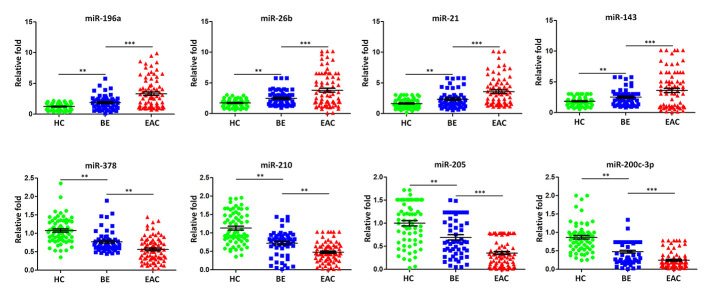
**Validation of miRNA array expression using independent samples.** TaqMan real-time RT-PCR to validate the expression levels of miR-196a, miR-26b, miR-21, miR-143, miR-378, miR-210, miR-205, and miR-200c-3pusing an independent cohort of 79 EAC patients, 56 BE patients and 66 healthy controls. Data shown are as mean ± SD.

To further validate the dysregulated exosomal miRNAs during BE-EAC sequential progression, TaqMan Real-Time PCR was employed to validate the differential expression levels of eight miRNAs including miR-196a, -26b, -21, -143, -378, -210, -205, and -200c-3p. These miRNA candidates were selected from the original microRNA assay screening according to their functional significance of previous investigations. An independent larger cohort of 79 EAC patients, 56 BE patients and 66 healthy controls was included here for further validation (Table 2). We found that exosomal miR-196a, -26b, -21, and -143 were significantly increased in individual BE patients when compared with healthy controls (Figure 3). In addition, the results demonstrated that exosomal miR-196a, -26b, -21, and -143 continued to have higher levels in EAC compared to BE (Figure 3); while miR-378, -210, -205, and -200c-3p were significantly under expressed in individual BE patients compared to healthy controls (Figure 3). Further exosomal miR-378, -210, -205, and -200c-3p continued to show lower levels in EAC patients (Figure 3). These findings confirmed that extracellular/circulating exosomal miRNAs are potentially important regulators influencing the sequential progression of Barrett’s esophagus to esophageal adenocarcinoma.

**Table 2:** Characteristics of independent cohorts of healthy controls and patients with BE or EAC.

**Variable**	**Controls (n = 66)**	**BE (n = 56)**	**EAC (n = 79)**
Gender (male)	36	32	45
Age (years, mean ± SD and range)	55±15	55±10	55±6
Barrett length (mean and range)	NA	C4M5 (C1M3–C10M11)	C8M8 (C5M5–C13M13)
Tumor stage (T)			
1	NA	NA	0
2			12
3			35
4			30
Unknown			2
Lymph node status (N)			
0	NA	NA	13
1			14
2			24
3			15
4			13
Unknown			0
Metastasis (M)			
0	NA	NA	33
1			40
Unknown			6
Body mass index (mean ± SD)	25±5	26±5	25±6
Current smokers	13	14	14
Previous smokers	22	14	15
Non-smokers	31	28	50

BE Barrett’s esophagus, EAC esophageal adenocarcinoma, CM circumference length, maximum length.

### MiRNAs targeting PD-L1 were down regulated in BE and EAC patients with PD-L1 positive expression

**Figure 4:**
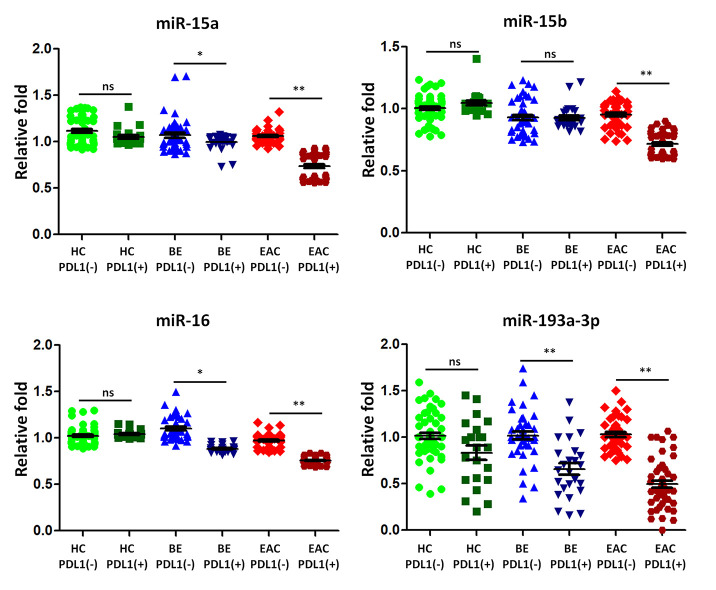
**The expression of microRNA predicted to target programmed death ligand 1 (PD-L1) is lower in BE and EAC with PD-L1 positivity.** TaqMan real-time RT-PCR to validate the expression levels of miR-15a, -15b, -16, and -193a-3p using the combined cohort of 25 BE and 48 EAC patients with PD-L1 positive samples compared to those PD-L1 negative patients (43 BE and 44 EAC). Healthy controls with positive PD-L1 patients (n=22) and negative PD-L1 patients (n=56) were also included for comparison. Data shown are as mean ± SD.

The upregulation of PD-L1 is found in cancers and contributes to evasion of the host immune defense. Some previous studies suggested that PD-L1 was regulated by miR-15a, -15b, -16, and -193a-3p targeting PD-L1 mRNA in human malignancies [31]. We analyzed the association between the expression of PD-L1 and that of sera exosomal miR-15a, -15b, -16, and -193a-3p in 25 BE and 48 EAC patients with PD-L1 positive expression compared to those PD-L1 negative patients (43 BE and 44 EAC). Healthy controls with positive PD-L1 individuals (n=22) and negative PD-L1 individuals (n=56) were also included for comparison. The median expression levels of sera exosomal miR-15a, -16, and -193a-3p were significantly down regulated (all p < 0.05) in BE PD-L1(+) patients when compared to BE PD-L1(-) patients (Figure 4); further sera exosomal miR-15a, -15b, -16, and -193a-3p expression levels in EAC PD-L1(+) patients were significantly lower (all p < 0.01) when compared to EAC PD-L1(-) patients (Figure 4). However, there were no significant differentially expressed between healthy controls with positive PD-L1 patients and negative PD-L1 patients (Figure 4).

### MiRNAs targeting PD-L1 were reduced expressed in BE patients with continually developing EAC

**Figure 5:**
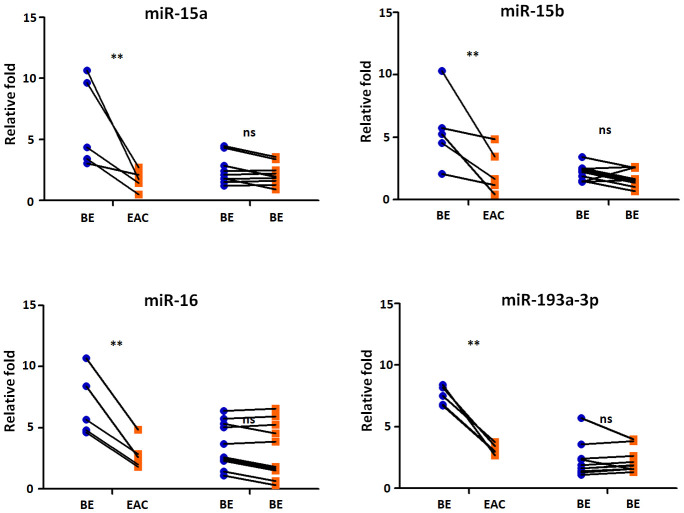
**Exosomal miRNAs expression correlates with BE-EAC progression.** TaqMan real-time RT-PCR to validate the expression levels of miR-15a, -15b, -16, and -193a-3p using 5 BE-EAC patients and 10 BE-BE patients. Data shown are as mean ± SD.

We then selected five BE patients continually developing EAC in 2 years from our cohorts, we tested the sera exosmal miRNAs expression of miR-15a, -15b, -16, and -193a-3p longitudinally. 10 BE patients who did not develop EAC in 5 years were included as control group. Compared with control group, the BE-EAC group had decreased sera exosomal expression levels of miR-15a, -15b, -16, and -193a-3p from BE status to their EAC progression (Figure 5), suggesting that these exosomal microRNAs could be early biomarkers for EAC development in BE patients. Furthermore, there were 3 PD-L1(-) and 2 PD-L1 (+) patients when BE status, all turned into PD-L1 (+) patients in all 5 BE patients who progressed to EAC, However, all samples were negative expression of PD-L1 status in 10 BE patients who did not progress to EAC. However, the statistic test is not significant at p < 0.05 due to the small sample size.

## DISCUSSION

BE patients are at a higher risk of developing EAC, which has the poor survival in its late stage [13]. In the current study, we performed a profiling of sera exosomal microRNA among BE and EAC patients. Distinct microRNA patterns were identified during the progression of BE to EAC. These differential exosomal miRNAs were validated with independent cohort, including miR-196a, -26b, -21, -143, -378, -210, -205, and -200c-3p. Expression of miR-196a, -26b, -21, and -143 were significantly higher in BE and continue to have higher levels in EAC compared to healthy controls; while miR-378, -210, -205, and -200c-3p were significantly lower expressed in BE patients compared to compared to controls. Further, miR-378, -210, -205, and -200c-3p continue to have even lower levels in EAC patients compared to BE. microRNAs-196a was found to correlate with during BE related dysplasia progression into esophagus adenocarcinoma [32]. Moreover, miR-196a levels were conversely correlated with long-term survival esophageal adenocarcinoma [33] and regulated the role of pro- and anti-apoptotic functions by targeting keratin 5, small prolinerich protein 2C, and S100 calcium-binding protein A9 [34]. MiR-21 was proven to be aberrantly increased in Barrett’s oesophagus [35], which was further enhanced by the activated EGFR signaling pathway in lung cancers [36]. Up regulation of miR-143 was found in BE and EAC [35], suggesting miR-143 may play an important role in dysplaysia and tumor develoment, possibly through targeting DNMT3A [37]. Taken all together, our findings strongly support potential roles of miRNAs in BE and sequential EAC tumorigenesis. We further confirmed exosomal miRNAs were differentially expressed at different stages of BE and EAC. We identified and assessed a miRNA combination that could detect the presence of EAC in at-risk BE patients (Figure 2C). The miRNA classifier is a potential biomarker for esophageal adenocarcinoma in patients at risk. The miRNA classifier could be valuable to detect preclinical esophageal adenocarcinoma, providing patients with a chance of curative resection and longer survival.

In addition, Fatty acid biosynthesis (2.238E-12), Hippo signaling pathway (2.604E-06), ErbB signaling pathway (1.001E-04) were identified with enrichment into the serum miRNA signature (Supplementary Table 1), indicating important biological signals coordinating the sequential progression of BE to EAC. Fatty acid synthase (FASN) as a key enzyme participated in the fatty acid biosynthetic pathway, and Over expression of FASN protein was observed in over half of BE patients, especially in the intestinal mucin phenotype of Barrett’s esophagus in which glandular cells display increased proliferation, angiogenesis, and COX-2 expression; Further FASN over expression pattern was retained in EAC [38]. Recent investigations demonstrated that amplification of *ERBB2* (also called *HER2*) oncogenic form was found in esophageal adenocarcinoma progression, leading to a worse outcome for EAC [39]. Combined with our current results, BE and EAC dysregulated miRNAs can regulate Fatty acid biosynthesis and ErbB signaling pathway, further contribute to development of EAC.

Up regulation of the surface expression of PD-L1 in tumor cells can interact with its receptor PD-1 on T cells, leading to evade immune surveillance and inhibit the immune checkpoint response [40]. The roles of microRNAs in transcriptional regulating the expression of PD-1/PD-L1 immune checkpoint remain elusive. In argonautes immunoprecipitation experiments, Kaso SC et al., discovered an interaction between the PD-L1 mRNA and the miR-15a or miR-16 [31]. Furthermore, Wang X et al., observed an inverse correlation between PD-L1 and miR-34a expression in acute myeloid leukemia [41]. Further, miR-93-5p and miR-106b-5p could target expression of PD-L1 mRNA in bulk cancer cells [42]. MiR-424 could regulate the PD-1/PD-L1 pathways in chemo resistant ovarian cancer through direct binding to PD-L1 mRNA 3’UTR [43]. In this study we for the first time found that microRNA expression levels of sera miR-15a, -16, and -193a-3p were significantly down regulated (all p < 0.05) in BE PD-L1(+) patients (Figure 5); further sera miR-15a, -15b, -16, and -193a-3p expression levels in EAC PD-L1(+) patients were significantly lower (all p < 0.01) when compared to EAC PD-L1(-) patients (Figure 5). These results demonstrated that tumor suppressor microRNAs may contribute to the regulation of PD-L1 expression in BE and EAC. More importantly, compared with control group, the BE-EAC group had decreased sera exosomal expression levels of miR-15a, -15b, -16, and -193a-3p from BE status to their EAC progression (Figure 5), suggesting that these exosomal microRNAs could be not only early biomarkers for EAC development in BE patients, further differential expressed microRNAs in Barrett’s esophagus may target PD-L1 expression and contribute to the development of esophageal adenocarcinoma.

It is deserved to search for changes of microRNA expression from BE to EAC as well as healthy subjects, and clarify the rising and decreasing groups of microRNA. Further investigation shows that microRNAs expression, which are likely to be involved in the progression of the parthenogenesis of EAC development from BE, compared the group that appears to have progressed in the same patient with the unchanged group. However, there are still some limitations in our study. BE and EAC samples were not paired in our study, but from separate individuals. In addition, we did not perform any functional investigation *in vivo* of the identified miRNAs. Another limitation for longitudinal analysis is that the sample size from the BE patients with continually developing EAC is small.

## CONCLUSIONS

In conclusion, here we performed a retrospective study by enrolling independent and large validation set of subjects, our results demonstrated that circulating exosomal miRNAs may have a promising diagnostic performance for the detection of BE and EAC. Further circulating exosomes contain differential level of microRNAs targeting PD-L1 mRNA in Barrett’s esophagus and esophageal adenocarcinoma.

## MATERIALS AND METHODS

### Patients

BE patients and EAC patients at Hospital, Tongji Medical College, Huazhong University of Science and Technology, Wuhan, China were enrolled between 2012 through 2019 after obtaining informed consent and approval by the Human Research Ethics Committee of Tongji Medical College, Huazhong University of Science and Technology. All participants in the first pilot study, including controls, underwent routine endoscopy [12 healthy controls, 12 BE and 13 EAC, Clinical features are displayed in Table 3]. Healthy subjects were patients undergoing endoscopy for unexplained upper abdominal complaints, had no reflux symptoms or endoscopic abnormalities. A further validation assay included an independent larger cohort of 79 EAC patients, 56 BE patients and 66 healthy controls. No significant difference of mean BMI between healthy controls, BE and EAC patients in both cohorts. The diagnoses of BE are made on the basis of histologic specimens evaluated by two specialized GI pathologists. Patients with concurrent or previous diagnosis of low-grade dysplasia (LGD), high-grade dysplasia (HGD), or EAC were excluded from BE patients in this study. Within this cohort, we also searched the cases that were further diagnosed to develop EAC during the follow-up period. There were totally 68 BE patients recruited in this study. The median follow-up time was 4.1 years (interquartile range, 1.5 to 7.5) for the 68 BE patients. Among them, 33 BE patients had more than 5 year follow up after they were first diagnosed as BE. The tissue microarray was applied to determine PD-L1 status in this study as described previously [44]. Briefly, immunohistochemistry was performed on a Leica Bond III platform using the Bond Polymer Refine Detection Kit (Leica Biosystems). The primary antibody against PD-L1 (SP142, rabbit IgG; Spring Bioscience, Pleasanton, CA) was incubated for 30 minutes at room temperature at a 1:50 dilution. This antibody had been previously validated in a large number of NSCLCs and was shown to be satisfactory (>95% concordance). For quantitative evaluation, an average score was derived by averaging the scores from each patient. Cases of PD-L1 expression of 5% or more were considered positive. Clinical data were retrieved from the hospital’s patient records. The experimental design and analysis were included in the workflow, as shown in Supplementary Figure 1.

**Table 3:** Characteristics of normal controls and patients with Barrett’s esophagus or esophageal adenocarcinoma used for microRNA expression profiling.

**Variable**	**Controls (n = 12)**	**BE (n = 12)**	**EAC (n = 13)**
Gender (male)	6	6	7
Age (years, mean ± SD and range)	52±13	53±9	51±16
Barrett length (mean and range)	NA	C4M5 (C1M3–C10M11)	C8M8 (C5M5–C13M13)
Tumor stage (T)			
1	NA	NA	0
2			4
3			4
4			5
Unknown			0
Lymph node status (N)			
0	NA	NA	2
1			2
2			4
3			3
4			2
Unknown			0
Metastasis (M)			
0	NA	NA	4
1			6
Unknown			3
Body mass index (mean ± SD)	26±6	25±4	25±3
Current smokers	2	3	1
Previous smokers	1	2	2
Non-smokers	9	7	10

BE Barrett’s esophagus, EAC esophageal adenocarcinoma, CM circumference length, maximum length.

### Exosome isolation from sera by precipitation

Exosomes from sera was extracted using total exosome isolation kit (Life technologies) [45]. Briefly, 15 ml serum was centrifuged at 3,000 g for 30 min and thereafter at 10,000 g for 30 min to remove cells and debris. Sera were further diluted with PBS and centrifuged at 10,000g for 10 min at room temperature. The pelleted exosomes was solubilized with PBS and utilized for characterization.

### Nanoparticle tracking analysis

Size distribution and concentration was analyzed using nanoparticle tracking analysis (NTA) on a NanoSight NS500 (Malvern Instruments Ltd.). Samples were recorded at camera level=14, detection level=3 and 5 videos of 20 seconds with a coefficient of variance <20% were used for analysis. To ensure measurementa ccuracy all samples were analyzed during the same time frame for each cohort/experiment on a single instrument by the same operator.

### Transmission electron microscopy (TEM)

Isolated exosomes were loaded onto formwar carbon-coated grids, fixed in 2% paraformaldehyde, washed and immunolabelled with anti-CD63 antibody followed by 10 nm gold-labelled secondary antibody (Sigma Aldrich). The exosomes were post-fixed in 2.5% glutaraldehyde, washed, contrasted in 2% uranyl acetate, embedded in a mixture of uranyl acetate (0.4%) and methyl cellulose (0.13%), and examined by electron microscope (Carl Zeiss NTS).

### MicroRNA expression profiling

Sera exosmal RNA was extracted using the miRNeasy Mini kit (Qiagen) following the manufacturer’s protocol. RNA quality was assessed by using a Nanodrop spectrophotometer (Thermo Scientific). 25 ng RNA was reverse transcribed for cDNA synthesis using the TaqMan Multiplex RT set (Applied Biosystems). qPCR was performed using TaqMan 2X Universal PCR Master Mix, No AmpErase UNG (Applied Biosystems). TaqMan Low-Density Array Human miRNA Panel (Applied Biosystems, Foster City, CA) was chosen for miRNA profiling.

### Pathway analysis

DIANA-mirPath [46] was employed to perform the enrichment analysis of predicted target genes by one or more miRNAs in biological pathways. The software performs an enrichment analysis of multiple miRNA target genes to all known KEGG pathways based on two algorithms, microT-CDS [47, 48] and miRTarBase [49]. The software is available at http://microrna.gr/mirpath.

### TaqMan miRNA qPCR

Sera exosome RNA was isolated from newly recruited cohort of 79 EAC, 56 BE and 66 healthy controls using Qiagen miRNeasy Serum/Plasma Kit (Qiagen, Valencia, CA). Individual miRNA analysis was performed using TaqMan miRNA assays (Applied Biosystems, Foster City, CA). A synthetic RNA spike-in, C. elegans miR-39 miRNA mimic, was added prior to cDNA synthesis as a control for variations in reverse transcription efficiency. The PCR amplifications were carried out by incubation for 10 min at 95°C, followed by 40 amplification cycles at 95°C for 10 sec and 60°C for 1 min. All reactions were performed in duplicate. miRNA with more than half of the Ct values > 35 per group were excluded from the analysis. Relative expression was calculated using the 2^-ΔΔCt^ method. The relative miRNA levels were normalized to internal controls miR-16-5p.

### Western blotting

Protein samples from lysed exosomes (5 μg) were resolved by sodium dodecyl sulphate-polyacrylamide gel electrophoresis (SDS-PAGE) (10-15%) and transferred to polyvinylidene fluoride membranes (Millipore). Blots were blocked 1hour with 5% milk in Tris-buffered saline (TBS) with 0.1% Tween-20, and blotted 1 hour with the following primary antibodies including CD63 (#106228D, Invitrogen), CD9 (#sc20048, Santa Cruz), CD81 (#10630D, Invitrogen), or cytochrome c (#556433, BD Pharmingen). After three washes with TBS/0.1% Tween-20, filters were incubated for 1h at room temperature with an HRP-conjugated secondary antibody before being revealed with ECL substrate (Pierce Biotechnology).

### Statistical analysis

Statistical analysis was performed with the GraphPad (San Diego, CA). Group comparisons were analyzed using Kruskal-Wallis or Student’ t test. Statistical data were expressed as mean ± SD. P value less than 0.05 was considered statistically significant. The microRNA classifier (MSC) test was performed by logistic regression analysis following previously reported standard operating procedures with fixed parameters [50]. The sensitivity, specificity, and area under the receiver operating characteristic curve (AUC) were used to evaluate the performance of the classifiers.

### Compliance with ethics guidelines

The study was performed in accordance with relevant guidelines and regulations, following the approval of the licensing committee of Tongji Medical College, Huazhong University of Science and Technology, and conformed to the tenets of the Declaration of Helsinki. Written informed consent was obtained from all patients with healthy subjects. We thank the participants of the study.

## Supplementary Material

Supplementary Figure 1

Supplementary Table 1

## References

[1] Cameron AJ, Souto EO, Smyrk TC. Small adenocarcinomas of the esophagogastric junction: association with intestinal metaplasia and dysplasia. Am J Gastroenterol. 2002; 97:1375–80. 10.1111/j.1572-0241.2002.05669.x 12094853

[2] Bennett C, Moayyedi P, Corley DA, DeCaestecker J, Falck-Ytter Y, Falk G, Vakil N, Sanders S, Vieth M, Inadomi J, Aldulaimi D, Ho KY, Odze R, et al, and BOB CAT Consortium. BOB CAT: a large-scale review and delphi consensus for management of barrett’s esophagus with no dysplasia, indefinite for, or low-grade dysplasia. Am J Gastroenterol. 2015; 110:662–82. 10.1038/ajg.2015.55 25869390 PMC4436697

[3] Rex DK, Cummings OW, Shaw M, Cumings MD, Wong RK, Vasudeva RS, Dunne D, Rahmani EY, Helper DJ. Screening for barrett’s esophagus in colonoscopy patients with and without heartburn. Gastroenterology. 2003; 125:1670–77. 10.1053/j.gastro.2003.09.030 14724819

[4] Ward EM, Wolfsen HC, Achem SR, Loeb DS, Krishna M, Hemminger LL, DeVault KR. Barrett’s esophagus is common in older men and women undergoing screening colonoscopy regardless of reflux symptoms. Am J Gastroenterol. 2006; 101:12–17. 10.1111/j.1572-0241.2006.00379.x 16405528

[5] Ronkainen J, Aro P, Storskrubb T, Johansson SE, Lind T, Bolling-Sternevald E, Vieth M, Stolte M, Talley NJ, Agréus L. Prevalence of barrett’s esophagus in the general population: an endoscopic study. Gastroenterology. 2005; 129:1825–31. 10.1053/j.gastro.2005.08.053 16344051

[6] Zagari RM, Fuccio L, Wallander MA, Johansson S, Fiocca R, Casanova S, Farahmand BY, Winchester CC, Roda E, Bazzoli F. Gastro-oesophageal reflux symptoms, oesophagitis and barrett’s oesophagus in the general population: the loiano-monghidoro study. Gut. 2008; 57:1354–59. 10.1136/gut.2007.145177 18424568

[7] Zou D, He J, Ma X, Chen J, Gong Y, Man X, Gao L, Wang R, Zhao Y, Yan X, Liu W, Wernersson B, Johansson S, et al. Epidemiology of symptom-defined gastroesophageal reflux disease and reflux esophagitis: the systematic investigation of gastrointestinal diseases in China (SILC). Scand J Gastroenterol. 2011; 46:133–41. 10.3109/00365521.2010.521888 20955088

[8] Falk GW, Thota PN, Richter JE, Connor JT, Wachsberger DM. Barrett's esophagus in women: demographic features and progression to high-grade dysplasia and cancer. Clin Gastroenterol Hepatol. 2005; 3:1089–94. 10.1016/s1542-3565(05)00606-3 16271339

[9] Shaheen NJ, Richter JE. Barrett’s oesophagus. Lancet. 2009; 373:850–61. 10.1016/S0140-6736(09)60487-6 19269522

[10] Schnell TG, Sontag SJ, Chejfec G, Aranha G, Metz A, O’Connell S, Seidel UJ, Sonnenberg A. Long-term nonsurgical management of barrett’s esophagus with high-grade dysplasia. Gastroenterology. 2001; 120:1607–19. 10.1053/gast.2001.25065 11375943

[11] Stein HJ, Siewert JR. Barrett’s esophagus: pathogenesis, epidemiology, functional abnormalities, Malignant degeneration, and surgical management. Dysphagia. 1993; 8:276–88. 10.1007/BF01354551 8359051

[12] Ye W, Chow WH, Lagergren J, Yin L, Nyrén O. Risk of adenocarcinomas of the esophagus and gastric cardia in patients with gastroesophageal reflux diseases and after antireflux surgery. Gastroenterology. 2001; 121:1286–93. 10.1053/gast.2001.29569 11729107

[13] Wang KK, Sampliner RE, Practice Parameters Committee of the American College of Gastroenterology. Updated guidelines 2008 for the diagnosis, surveillance and therapy of barrett’s esophagus. Am J Gastroenterol. 2008; 103:788–97. 10.1111/j.1572-0241.2008.01835.x 18341497

[14] Vrána D, Matzenauer M, Neoral Č, Aujeský R, Vrba R, Melichar B, Rušarová N, Bartoušková M, Jankowski J. From tumor immunology to immunotherapy in gastric and esophageal cancer. Int J Mol Sci. 2018; 20:13. 10.3390/ijms20010013 30577521 PMC6337592

[15] Alsina M, Moehler M, Lorenzen S. Immunotherapy of esophageal cancer: current status, many trials and innovative strategies. Oncol Res Treat. 2018; 41:266–71. 10.1159/000488120 29705786

[16] Fan J, Yin Z, Xu J, Wu F, Huang Q, Yang L, Jin Y, Yang G. Circulating microRNAs predict the response to anti-PD-1 therapy in non-small cell lung cancer. Genomics. 2020; 112:2063–71. 10.1016/j.ygeno.2019.11.019 31786291

[17] Yoon YJ, Kim OY, Gho YS. Extracellular vesicles as emerging intercellular communicasomes. BMB Rep. 2014; 47:531–39. 10.5483/bmbrep.2014.47.10.164 25104400 PMC4261509

[18] Willms E, Johansson HJ, Mäger I, Lee Y, Blomberg KE, Sadik M, Alaarg A, Smith CI, Lehtiö J, El Andaloussi S, Wood MJ, Vader P. Cells release subpopulations of exosomes with distinct molecular and biological properties. Sci Rep. 2016; 6:22519. 10.1038/srep22519 26931825 PMC4773763

[19] Bartel DP. MicroRNAs: genomics, biogenesis, mechanism, and function. Cell. 2004; 116:281–97. 10.1016/s0092-8674(04)00045-5 14744438

[20] Iorio MV, Croce CM. MicroRNA dysregulation in cancer: diagnostics, monitoring and therapeutics. A comprehensive review. EMBO Mol Med. 2012; 4:143–59. 10.1002/emmm.201100209 22351564 PMC3376845

[21] Iorio MV, Croce CM. microRNA involvement in human cancer. Carcinogenesis. 2012; 33:1126–33. 10.1093/carcin/bgs140 22491715 PMC3514864

[22] Croce CM. Causes and consequences of microRNA dysregulation in cancer. Nat Rev Genet. 2009; 10:704–14. 10.1038/nrg2634 19763153 PMC3467096

[23] Chiam K, Wang T, Watson DI, Mayne GC, Irvine TS, Bright T, Smith L, White IA, Bowen JM, Keefe D, Thompson SK, Jones ME, Hussey DJ. Circulating Serum Exosomal miRNAs As Potential Biomarkers for Esophageal Adenocarcinoma. J Gastrointest Surg. 2015; 19:1208–15. 10.1007/s11605-015-2829-9 25943911

[24] Matsuzaki J, Suzuki H. MicroRNAs in barrett’s esophagus: future prospects. Front Genet. 2014; 5:69. 10.3389/fgene.2014.00069 24765103 PMC3982049

[25] Hemmatzadeh M, Mohammadi H, Karimi M, Musavishenas MH, Baradaran B. Differential role of microRNAs in the pathogenesis and treatment of Esophageal cancer. Biomed Pharmacother. 2016; 82:509–19. 10.1016/j.biopha.2016.05.009 27470391

[26] Mallick R, Patnaik SK, Wani S, Bansal A. A systematic review of esophageal MicroRNA markers for diagnosis and monitoring of barrett’s esophagus. Dig Dis Sci. 2016; 61:1039–50. 10.1007/s10620-015-3959-3 26572780

[27] Matsui D, Zaidi AH, Martin SA, Omstead AN, Kosovec JE, Huleihel L, Saldin LT, DiCarlo C, Silverman JF, Hoppo T, Finley GG, Badylak SF, Kelly RJ, Jobe BA. Primary tumor microRNA signature predicts recurrence and survival in patients with locally advanced esophageal adenocarcinoma. Oncotarget. 2016; 7:81281–91. 10.18632/oncotarget.12832 27793030 PMC5348392

[28] Yang H, Gu J, Wang KK, Zhang W, Xing J, Chen Z, Ajani JA, Wu X. MicroRNA expression signatures in Barrett's esophagus and esophageal adenocarcinoma. Clin Cancer Res. 2009; 15:5744–52. 10.1158/1078-0432.CCR-09-0385 19737949 PMC2745487

[29] Smith CM, Watson DI, Michael MZ, Hussey DJ. MicroRNAs, development of barrett’s esophagus, and progression to esophageal adenocarcinoma. World J Gastroenterol. 2010; 16:531–37. 10.3748/wjg.v16.i5.531 20128019 PMC2816263

[30] Kan T, Meltzer SJ. MicroRNAs in barrett’s esophagus and esophageal adenocarcinoma. Curr Opin Pharmacol. 2009; 9:727–32. 10.1016/j.coph.2009.08.009 19773200 PMC2794797

[31] Kao SC, Cheng YY, Williams M, Kirschner MB, Madore J, Lum T, Sarun KH, Linton A, McCaughan B, Klebe S, van Zandwijk N, Scolyer RA, Boyer MJ, et al. Tumor suppressor microRNAs contribute to the regulation of PD-L1 expression in Malignant pleural mesothelioma. J Thorac Oncol. 2017; 12:1421–33. 10.1016/j.jtho.2017.05.024 28629895

[32] Luzna P, Gregar J, Uberall I, Radova L, Prochazka V, Ehrmann J Jr. Changes of microRNAs-192, 196a and 203 correlate with barrett’s esophagus diagnosis and its progression compared to normal healthy individuals. Diagn Pathol. 2011; 6:114. 10.1186/1746-1596-6-114 22094011 PMC3268741

[33] Bloomston M, Frankel WL, Petrocca F, Volinia S, Alder H, Hagan JP, Liu CG, Bhatt D, Taccioli C, Croce CM. MicroRNA expression patterns to differentiate pancreatic adenocarcinoma from normal pancreas and chronic pancreatitis. JAMA. 2007; 297:1901–08. 10.1001/jama.297.17.1901 17473300

[34] Maru DM, Singh RR, Hannah C, Albarracin CT, Li YX, Abraham R, Romans AM, Yao H, Luthra MG, Anandasabapathy S, Swisher SG, Hofstetter WL, Rashid A, Luthra R. MicroRNA-196a is a potential marker of progression during barrett’s metaplasia-dysplasia-invasive adenocarcinoma sequence in esophagus. Am J Pathol. 2009; 174:1940–48. 10.2353/ajpath.2009.080718 19342367 PMC2671281

[35] Fassan M, Volinia S, Palatini J, Pizzi M, Baffa R, De Bernard M, Battaglia G, Parente P, Croce CM, Zaninotto G, Ancona E, Rugge M. MicroRNA expression profiling in human barrett’s carcinogenesis. Int J Cancer. 2011; 129:1661–70. 10.1002/ijc.25823 21128279 PMC4303574

[36] Seike M, Goto A, Okano T, Bowman ED, Schetter AJ, Horikawa I, Mathe EA, Jen J, Yang P, Sugimura H, Gemma A, Kudoh S, Croce CM, Harris CC. MiR-21 is an EGFR-regulated anti-apoptotic factor in lung cancer in never-smokers. Proc Natl Acad Sci USA. 2009; 106:12085–90. 10.1073/pnas.0905234106 19597153 PMC2715493

[37] Shen JZ, Zhang YY, Fu HY, Wu DS, Zhou HR. Overexpression of microRNA-143 inhibits growth and induces apoptosis in human leukemia cells. Oncol Rep. 2014; 31:2035–42. 10.3892/or.2014.3078 24626955

[38] Ishimura N, Amano Y, Sanchez-Siles AA, Fukuhara H, Takahashi Y, Uno G, Tamagawa Y, Mishima Y, Yuki T, Ishihara S, Kinoshita Y. Fatty acid synthase expression in barrett’s esophagus: implications for carcinogenesis. J Clin Gastroenterol. 2011; 45:665–72. 10.1097/MCG.0b013e318207f240 21325951

[39] Walch A, Bink K, Hutzler P, Höfler H, Werner M. HER-2/neu gene amplification by FISH predicts poor survival in barrett’s esophagus-associated adenocarcinoma. Hum Pathol. 2000; 31:1332–34. 11070129

[40] Chen L, Han X. anti-PD-1/PD-L1 therapy of human cancer: past, present, and future. J Clin Invest. 2015; 125:3384–91. 10.1172/JCI80011 26325035 PMC4588282

[41] Wang X, Li J, Dong K, Lin F, Long M, Ouyang Y, Wei J, Chen X, Weng Y, He T, Zhang H. Tumor suppressor miR-34a targets PD-L1 and functions as a potential immunotherapeutic target in acute myeloid leukemia. Cell Signal. 2015; 27:443–52. 10.1016/j.cellsig.2014.12.003 25499621

[42] Cioffi M, Trabulo SM, Vallespinos M, Raj D, Kheir TB, Lin ML, Begum J, Baker AM, Amgheib A, Saif J, Perez M, Soriano J, Desco M, et al. The miR-25-93-106b cluster regulates tumor metastasis and immune evasion via modulation of CXCL12 and PD-L1. Oncotarget. 2017; 8:21609–25. 10.18632/oncotarget.15450 28423491 PMC5400610

[43] Xu S, Tao Z, Hai B, Liang H, Shi Y, Wang T, Song W, Chen Y, OuYang J, Chen J, Kong F, Dong Y, Jiang SW, et al. miR-424(322) reverses chemoresistance via t-cell immune response activation by blocking the PD-L1 immune checkpoint. Nat Commun. 2016; 7:11406. 10.1038/ncomms11406 27147225 PMC4858750

[44] Gradecki SE, Grange JS, Stelow EB. Concordance of PD-L1 expression between core biopsy and resection specimens of non-small cell lung cancer. Am J Surg Pathol. 2018; 42:1090–94. 10.1097/PAS.0000000000001085 29794870

[45] Freeman DW, Noren Hooten N, Eitan E, Green J, Mode NA, Bodogai M, Zhang Y, Lehrmann E, Zonderman AB, Biragyn A, Egan J, Becker KG, Mattson MP, et al. Altered extracellular vesicle concentration, cargo, and function in diabetes. Diabetes. 2018; 67:2377–88. 10.2337/db17-1308 29720498 PMC6198336

[46] Papadopoulos GL, Alexiou P, Maragkakis M, Reczko M, Hatzigeorgiou AG. DIANA-mirPath: integrating human and mouse microRNAs in pathways. Bioinformatics. 2009; 25:1991–93. 10.1093/bioinformatics/btp299 19435746

[47] Reczko M, Maragkakis M, Alexiou P, Grosse I, Hatzigeorgiou AG. Functional microRNA targets in protein coding sequences. Bioinformatics. 2012; 28:771–76. 10.1093/bioinformatics/bts043 22285563

[48] Paraskevopoulou MD, Georgakilas G, Kostoulas N, Vlachos IS, Vergoulis T, Reczko M, Filippidis C, Dalamagas T, Hatzigeorgiou AG. DIANA-microT web server v5.0: service integration into miRNA functional analysis workflows. Nucleic Acids Res. 2013; 41:W169–73. 10.1093/nar/gkt393 23680784 PMC3692048

[49] Hsu SD, Lin FM, Wu WY, Liang C, Huang WC, Chan WL, Tsai WT, Chen GZ, Lee CJ, Chiu CM, Chien CH, Wu MC, Huang CY, et al. miRTarBase: a database curates experimentally validated microRNA-target interactions. Nucleic Acids Res. 2011; 39:D163–9. 10.1093/nar/gkq1107 21071411 PMC3013699

[50] Mensah M, Borzi C, Verri C, Suatoni P, Conte D, Pastorino U, Orazio F, Sozzi G, Boeri M. MicroRNA based liquid biopsy: the experience of the plasma miRNA signature classifier (MSC) for lung cancer screening. J Vis Exp. 2017; 56326. 10.3791/56326 29155727 PMC5755225

