# Supplementary figures and images for: Evidence that dysplasia related microRNAs in Barrett’s esophagus target PD-L1 expression and contribute to the development of esophageal adenocarcinoma
Source: Aging (Albany NY). 2020 Sep 9;12(17):17062–78. doi: 10.18632/aging.103634 (PMC7521496; doi:10.18632/aging.103634)

SUPPLEMENTARY FIGURE

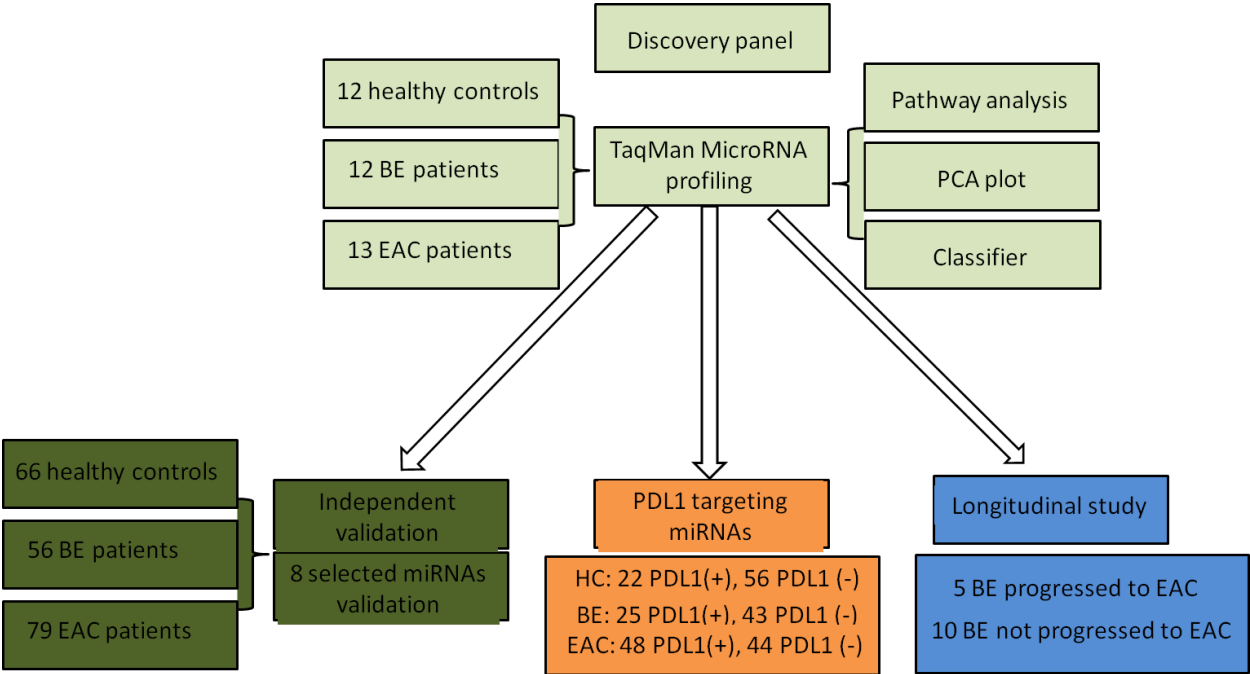

Supplementary Figure 1. The workflow in this study.

Supplement: Supplementary Figure 1 [file aging-12-103634-s002..pdf]
