# Supplementary material for: Evidence that dysplasia related microRNAs in Barrett’s esophagus target PD-L1 expression and contribute to the development of esophageal adenocarcinoma
Source: Aging (Albany NY). 2020 Sep 9;12(17):17062–78. doi: 10.18632/aging.103634 (PMC7521496; doi:10.18632/aging.103634)
Supplement: Supplementary Table 1 [file aging-12-103634-s001..pdf]

SUPPLEMENTARY TABLE

Supplementary Table 1. Biologic pathways enriched by differentially expressed serum miRNAs.

KEGG pathway	p-value	#genes	#miRNAs
Top of Form<br>Fatty acid biosynthesis (hsa00061) Bottom of Form	2.238E-12	Top of Form<br>7Bottom of Form	6
Top of Form<br>Hippo signaling pathway (hsa04390) Bottom of Form	2.604E-06	Top of Form<br>70Bottom of Form	15
Top of Form<br>Axon guidance (hsa04360) Bottom of Form	2.604E-06	Top of Form<br>69Bottom of Form	16
Top of Form<br>Proteoglycans in cancer (hsa05205)Bottom of Form	3.331E-05	Top of Form<br>93Bottom of Form	18
Top of Form<br>Glioma (hsa05214) Bottom of Form	5.042E-05	Top of Form<br>35Bottom of Form	15
Top of Form<br>Adrenergic signaling in cardiomyocytes(hsa04261) Bottom of Form	9.324E-05	Top of Form<br>69Bottom of Form	17
Top of Form<br>Neurotrophin signaling pathway (hsa04722) Bottom of Form	9.324E-05	Top of Form<br>65Bottom of Form	18
Top of Form<br>ErbB signaling pathway (hsa04012)Bottom of Form	1.001E-04	Top of Form<br>50Bottom of Form	18
Top of Form<br>Glutamatergic synapse (hsa04724) Bottom of Form	1.972E-04	Top of Form<br>53Bottom of Form	15
Top of Form<br>Oxytocin signaling pathway (hsa04921)Bottom of Form	2.029E-04	Top of Form<br>79Bottom of Form	16
Top of Form<br>FoxO signaling pathway (hsa04068) Bottom of Form	2.363E-04	Top of Form<br>67Bottom of Form	16
Top of Form<br>Long-term potentiation (hsa04720) Bottom of Form	4.527E-04	Top of Form<br>39Bottom of Form	15
Top of Form<br>cGMP-PKG signaling pathway (hsa04022) Bottom of Form	5.394E-04	Top of Form<br>79Bottom of Form	18
Top of Form<br>mTOR signaling pathway (hsa04150) Bottom of Form	7.141E-04	Top of Form<br>35Bottom of Form	15
Top of Form<br>Wnt signaling pathway (hsa04310) Bottom of Form	7.141E-04	Top of Form<br>66Bottom of Form	16
Top of Form<br>Signaling pathways regulating pluripotency of stem cells (hsa04550) Bottom of Form	7.141E-04	Top of Form<br>67Bottom of Form	18
Top of Form<br>PI3K-Akt signaling pathway (hsa04151) Bottom of Form	7.141E-04	Top of Form<br>146Bottom of Form	19
Top of Form<br>Acute myeloid leukemia (hsa05221) Bottom of Form	1.070E-03	Top of Form<br>33Bottom of Form	15
Top of Form<br>Long-term depression (hsa04730) Bottom of Form	1.318E-03	Top of Form<br>31Bottom of Form	11
Top of Form<br>Ras signaling pathway (hsa04014) Bottom of Form	1.318E-03	Top of Form<br>100Bottom of Form	18
Top of Form<br>Amphetamine addiction (hsa05031)Bottom of Form	1.354E-03	Top of Form<br>33Bottom of Form	16
Top of Form<br>Prostate cancer (hsa05215) Bottom of Form	1.373E-03	Top of Form<br>46Bottom of Form	15

Top of Form<br>Pathways in cancer (hsa05200) Bottom of Form	1.373E-03	Top of Form<br>167Bottom of Form	18
Top of Form<br>Gap junction (hsa04540) Bottom of Form	1.412E-03	Top of Form<br>39Bottom of Form	15
Top of Form<br>Thyroid hormone signaling pathway (hsa04919) Bottom of Form	1.412E-03	Top of Form<br>53Bottom of Form	19
Top of Form<br>Glycosaminoglycan biosynthesis (hsa00534) Bottom of Form	1.864E-03	Top of Form<br>11Bottom of Form	10
Top of Form<br>Non-small cell lung cancer (hsa05223) Bottom of Form	1.864E-03	Top of Form<br>28Bottom of Form	15
Top of Form<br>Rap1 signaling pathway (hsa04015) Bottom of Form	1.864E-03	Top of Form<br>94Bottom of Form	19
Top of Form<br>AMPK signaling pathway (hsa04152) Bottom of Form	2.614E-03	Top of Form<br>60Bottom of Form	18
Top of Form<br>MAPK signaling pathway (hsa04010) Bottom of Form	3.176E-03	Top of Form<br>113Bottom of Form	19
